# Emergence of Monkeypox — West and Central Africa, 1970–2017

**DOI:** 10.15585/mmwr.mm6710a5

**Published:** 2018-03-16

**Authors:** Kara N. Durski, Andrea M. McCollum, Yoshinori Nakazawa, Brett W. Petersen, Mary G. Reynolds, Sylvie Briand, Mamoudou Harouna Djingarey, Victoria Olson, Inger K. Damon, Asheena Khalakdina

**Affiliations:** ^1^World Health Organization, Geneva, Switzerland; ^2^Division of High-Consequence Pathogens and Pathology, CDC; ^3^World Health Organization, Brazzaville, Republic of the Congo.

The recent apparent increase in human monkeypox cases across a wide geographic area, the potential for further spread, and the lack of reliable surveillance have raised the level of concern for this emerging zoonosis. In November 2017, the World Health Organization (WHO), in collaboration with CDC, hosted an informal consultation on monkeypox with researchers, global health partners, ministries of health, and orthopoxvirus experts to review and discuss human monkeypox in African countries where cases have been recently detected and also identify components of surveillance and response that need improvement. Endemic human monkeypox has been reported from more countries in the past decade than during the previous 40 years. Since 2016, confirmed cases of monkeypox have occurred in Central African Republic, Democratic Republic of the Congo, Liberia, Nigeria, Republic of the Congo, and Sierra Leone and in captive chimpanzees in Cameroon. Many countries with endemic monkeypox lack recent experience and specific knowledge about the disease to detect cases, treat patients, and prevent further spread of the virus. Specific improvements in surveillance capacity, laboratory diagnostics, and infection control measures are needed to launch an efficient response. Further, gaps in knowledge about the epidemiology and ecology of the virus need to be addressed to design, recommend, and implement needed prevention and control measures.

## Monkeypox Cases in West Africa and Central Africa

Since the global eradication of smallpox, monkeypox has emerged as the most prevalent orthopoxvirus infection in humans (*1*). The majority of documented human monkeypox cases have occurred in Democratic Republic of the Congo (DRC), where it was first recognized as a human disease in 1970; however, during the last decade, the number of cases in other west and central African countries have been increasing; many of these countries had not reported a case for several decades (Table) (Figure). Since 2016, monkeypox cases have been reported and confirmed from Central African Republic (19 cases), DRC (>1,000 reported per year), Liberia (two), Nigeria (>80), Republic of the Congo (88), and Sierra Leone (one) (Table); an outbreak in captive chimpanzees occurred in Cameroon. With 80 confirmed cases, Nigeria is currently experiencing the largest documented outbreak of human monkeypox in West Africa. The emergence of cases is a concern for global health security.

**TABLE:** Reported cases of monkeypox in humans and animals, by country — Africa,* 1970–2018

Country	Year	Location	No. of cases^†^	No. of deaths
Cameroon^§^	1979	Mfou District	1	0
	1989	Nkoteng	1	0
Central African Republic	1984	Sangha Administrative Region	6	0
	2001	—	4	—
	2010	—	2	0
	2015	Mbomou Prefecture, Bakouma and Bangassou subprefectures	12	3
	2016	Haute-Kotto Health District, Yalinga	11	1
	2017	Mbaiki Health District	2	0
	2017	Ouango Health Districts	6	0
Côte d’Ivoire^¶^	1971	Abengourou	1	0
	1981	—	1	—
Democratic Republic of the Congo	1970–2017	Multiple provinces	>1,000/year**	—
Gabon	1987	Region between Lambarene and N'Djole	5	2
Liberia	1970	Grand Geddah	4	0
	2017	Rivercess and Maryland counties	2	0
Nigeria	1971	Aba State	2	0
	1978	Oyo State	1	0
	2017–2018	Multiple states	89^††^	6^††^
Republic of the Congo	2003	Likouala Region	11	1
	2009	Likouala Region	2	0
	2017	Likouala Region	88	6
Sierra Leone	1970	Aguebu	1	0
	2014	Bo	1	1
	2017	Pujehan District	1	0
Sudan^§§,¶¶^	2005	Unity State	19	0

* The United States experienced a monkeypox outbreak in 2003 with 47 confirmed and probable cases, attributed to a shipment of wild animals from West Africa to the United States.

^†^ Includes laboratory-confirmed cases and suspected cases that had an epidemiologic (close contact), spatial, or temporal link to a laboratory-confirmed case.

^§^ Outbreaks have occurred twice (2014 and 2016) in captive chimpanzee groups.

^¶^ Monkeypox virus was isolated from a wild caught sooty mangabey (*Cercocebus atys*).

** Democratic Republic of the Congo has reported >1,000 suspected cases each year since 2005.

^††^ As of February 25, 2018; laboratory-confirmed cases only.

^§§^ The presence of Monkeypox virus in Sudan was attributed to movement of the virus from Democratic Republic of the Congo.

^¶¶^ The cases occurred in an area that is now part of South Sudan.

**FIGURE:**
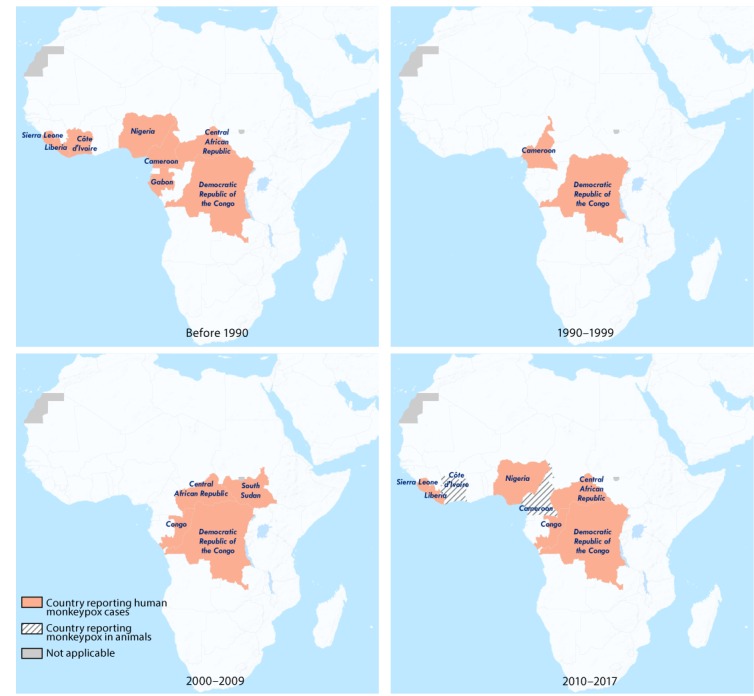
Countries reporting monkeypox cases in humans and animals — West and Central Africa, 1970–2017* * Current as of February 25, 2018.

Monkeypox is a zoonotic orthopoxvirus with a similar disease presentation to smallpox in humans, with the additional distinguishing symptom of lymphdenopathy. After an initial febrile prodrome, a centrifugally distributed maculopapular rash develops, with lesions often present on the palms of the hands and soles of the feet. The infection can last up to 4 weeks, until crusts separate and a fresh layer of skin is formed. Sequelae include secondary bacterial infections, respiratory distress, bronchopneumonia, gastrointestinal involvement, dehydration, encephalitis, and ocular infections, which can result in permanent corneal scarring. No specific treatment for a monkeypox virus infection currently exists, and patients are managed with supportive care and symptomatic treatment. In persons who have not been vaccinated against smallpox, which offers cross-protection, the case fatality rate is 11%. Human-to-human transmission occurs via respiratory droplets and contact with lesions that contain the virus (*1*).

Monkeypox primarily occurs in the rain forests in West Africa and Central Africa. Although antibodies have been detected in a range of small mammal species (*2*), the reservoir species of monkeypox remains unknown, and the virus has been isolated only twice from wild animals, once from a rope squirrel (*Funisciurus anerythrus*) in DRC and once from a sooty mangabey (*Cercocebus atys*) in Côte d’Ivoire. Contact with the animal reservoir/reservoirs, including contact with live or dead animals, often through the hunting and preparation of bushmeat as food, is a presumed driver of monkeypox infection. Closer contact between humans and animals through deforestation, demographic changes, climate change, hunting, and population movement might account for the recent increase in reported cases and expansion of geographic range. Civil war and population displacement can force inhabitants to seek alternative sources of protein, including the consumption of monkeys, squirrels, and other rodents.

Vaccination against smallpox is known to be cross-protective against the other orthopoxviruses, including monkeypox. Following the eradication of smallpox in 1980 and the cessation of smallpox vaccination in the early 1980s, waning vaccine-induced population immunity and lack of protection among younger age groups might have contributed to the resurgence of the disease (*3*).

Monkeypox virus has two recognized clades: West African and Congo Basin. Differences in epidemiologic and clinical features between viral isolates support the distinction between these two clades (*4*). Advances in the use of DNA sequencing to understand viral strains and populations will be valuable for interpreting transmission events and confirming the existence of endemic variants (*5*,*6*). Further studies are needed to understand temporal and spatial genetic differences in viral strains.

## Discussion

Monkeypox presents challenges for public health officials and health care personnel in terms of surveillance and laboratory capacities, and management and treatment of disease. Overall, surveillance in West Africa has improved as a result of recommendations from the Joint External Evaluations* and the Global Health Security Agenda assessments after the 2014–2016 Ebola virus disease epidemic. However, health care providers in many countries lack knowledge and experience in the recognition, diagnosis, and treatment of monkeypox, and implementation of public health measures that are needed to stop further spread. The establishment of appropriate disease surveillance systems requires initial and long-term financial and human resource investments. Monkeypox is not currently a disease for which mandatory reporting is required through the Integrated Disease Surveillance and Response system across Africa.^†^ DRC has implemented mandatory reporting of the disease, which has improved systematic reporting. Although notifications occur regularly, investigations with diagnostic specimens and implementation of control measures, including contact tracing and strict patient isolation, are less rigorously applied. Because monkeypox is a viral zoonosis, coordination of interventions between the human and animal (wildlife) health sectors is necessary, including routine sharing of information.

Laboratory confirmation of infection is critical, because human monkeypox closely resembles several other febrile rash illnesses including smallpox and varicella. The appropriate specimens for identification of the virus in active cases of monkeypox are swabs or crusts of lesions, in contrast to blood, serum, and sputum specimens collected by clinicians and laboratory technicians for diagnosis of many other diseases, and specimens must be accompanied by detailed clinical information for appropriate interpretation of laboratory results. Implementation of monkeypox-specific case investigation forms, and training health care workers in their use, can support appropriate case investigation and confirmation (*7*). The most efficient means of laboratory confirmation is through molecular assays, which will require strengthening of national laboratory capacity in countries with endemic disease. Regional and global reference laboratory systems need to be established to support diagnostic assay quality assurance and confirmation, and appropriate storage and safe transport of specimens in areas with limited infrastructure will require innovative solutions.

Monkeypox cases frequently occur in forested rural areas, which often have limited access to health services. The provision of clinical supportive care and treatment for complications such as ocular and secondary infections, respiratory involvement, and fluid imbalance, can be challenging because of resource and specialized care limitations (*7*,*8*).

Although infection prevention and control techniques and supplies are often lacking in rural areas, measures such as contact precautions, appropriate disinfection, and limited contact with patients can be implemented at health care facilities and patient homes. Patients and their families might also face stigma in their communities because of lack of knowledge about the disease and fear that cases might represent an epidemic such as Ebola, and rumors can cause panic; however, psychosocial support for patients and their families is often not prioritized. Education and risk communication for affected families and communities are important components of a public health response that addresses potentially risky behaviors, such as hunting and consumption of bushmeat and contact with ill persons. Engaging communities in developing feasible interventions and encouraging needed health-seeking behavior is important. If resources are available, contacts could be followed to limit further community exposures and halt subsequent chains of transmission. Information on final outcomes and long-term sequelae need to be better documented to improve understanding of the disease course (*8*).

Better collaboration between human and animal health personnel is needed to understand the impact of monkeypox among humans and animals and the mechanisms of animal-to-human transmission and to implement adequate prevention and response measures. Developing integrated, regional plans and ensuring cross-border coordination among countries that share geographically contiguous risk zones are needed to stop the spread of disease.

The 2018 list of priority diseases for the WHO Research and Development Blueprint identified monkeypox as an emerging disease requiring rapid evaluation of available potential countermeasures (*9*). In this regard, vaccines and medical therapeutics developed for smallpox could be validated for use against human monkeypox in clinical studies through operational research in countries with endemic disease to optimize their potential impact.

The increase in number of monkeypox cases being reported from countries in Africa that have not reported cases in several decades and the myriad factors that affect monkeypox transmission highlight the need to update knowledge about the disease and strengthen preparedness efforts. To address gaps in knowledge and expertise in areas with endemic disease, a number of areas of work are being prioritized by WHO in collaboration with CDC. To improve understanding of mechanisms of virus transmission, both zoonotic and interhuman, national disease surveillance systems need to be strengthened for humans, as well as for wildlife, using community-based event reporting. In countries with endemic disease, this includes the reporting of all suspected cases through the Integrated Disease Surveillance and Response system, collection of relevant disease-specific data to support laboratory diagnostic and epidemiological interpretation, and follow-up of confirmed cases.

Improvements in laboratory capacity require training in laboratory procedures, the types of specimens to collect, and safe specimen collection, storage, and transportation. Improvements in the capacity to detect monkeypox virus have been found to increase zoonotic disease detection and response, as seen during the Ebola virus disease response in Tshuapa Province of DRC (*10*). Regional trainings to increase national-level expertise and the sharing of country-level experiences will have the potential to build a network for exchange of best practices and technical support. Global health security will benefit from additional efforts to build regional-level capacity.

Including local-level training in national response and surveillance plans is important to ensure that health care workers and surveillance staff members in regions with endemic disease are equipped to detect and manage cases. In all these endeavors, WHO and orthopoxvirus reference centers such as CDC, Institut Pasteur Dakar (Senegal), and Institut National de Recherche Biomedicale (DRC) are working to provide guidance and technical support for the required public health actions.

As with all zoonotic diseases, a comprehensive One Health^§^ approach is necessary for disease detection and response, including wildlife surveillance and investigations into the animal reservoir/reservoirs, which require dedicated resources. Multicountry collaborations are important for sharing experiences, developing stronger national and regional capacities, and alerting neighboring countries of cases of monkeypox in humans and animals. Unlike smallpox, a human disease with no animal reservoir that was eradicated through vaccination campaigns, monkeypox has an animal reservoir/reservoirs. Insights into the animal reservoir and ecological niche will enable monitoring the virus’s movements outside the natural ecological setting. Improving understanding of monkeypox will aid in developing innovative solutions to mitigate further spread of the virus. Furthermore, improved detection and response capacity for monkeypox will enhance capacity for responding to other zoonoses and orthopoxvirus events at regional and national levels.

**Summary**

**What is already known about this topic?**

Human monkeypox is a viral zoonosis that occurs in West Africa and Central Africa. Most cases are reported from Democratic Republic of the Congo. The disease causes significant morbidity and mortality, and no specific treatment exists.

**What is added by this report?**

Nigeria is currently experiencing the largest documented outbreak of human monkeypox in West Africa. During the past decade, more human monkeypox cases have been reported in countries that have not reported disease in several decades. Since 2016, cases have been confirmed in Central African Republic (19 cases), Democratic Republic of the Congo (>1,000 reported per year), Liberia (two), Nigeria (>80), Republic of the Congo (88), and Sierra Leone (one). The reemergence of monkeypox is a global health security concern.

**What are the implications for public health practice?**

A recent meeting of experts and representatives from affected countries identified challenges and proposed actions to improve response actions and surveillance. The World Health Organization and CDC are developing updated guidance and regional trainings to improve capacity for laboratory-based surveillance, detection, and prevention of monkeypox, improved patient care, and outbreak response.

## Footnotes

* http://www.who.int/ihr/procedures/mission-reports-africa/en/.

† http://www.afro.who.int/publications/technical-guidelines-integrated-disease-surveillance-and-response-african-region-0.

§ https://www.cdc.gov/onehealth/index.html.
