# *Erratum*: Vol. 67, No. 10

**DOI:** 10.15585/mmwr.mm6716a7

**Published:** 2018-04-27

In the report “Emergence of Monkeypox — West and Central Africa, 1970–2017,” on page 306, the next to the last sentence in the first paragraph under “Monkeypox Cases in West Africa and Central Africa” should have read “With **89** confirmed cases, Nigeria is currently experiencing the largest documented outbreak of human monkeypox in West Africa.”

